# Effects of atropine on choroidal thickness in myopic children: a meta-analysis

**DOI:** 10.3389/fphar.2024.1440180

**Published:** 2024-10-21

**Authors:** Yaqi Yang, Lijuan Wei, Bo Wang, Wei Zheng

**Affiliations:** Ophthalmology Department, Affiliated Hospital of Changchun University of Traditional Chinese Medicine, Changchun, China

**Keywords:** atropine, choroidal thickness, myopia, AL, SER

## Abstract

**Background:**

Atropine is an effective medicine for myopia prevention and control. This meta-analysis was conducted to investigate the effects of atropine on choroidal thickness (ChT) in children with myopia.

**Methods:**

Between its inception and 1 June 2023, Medline, Embase, and Web of Science were all searched, and only English literature was included. The choroidal thickness was the primary study outcome. Axial length, standardized equivalent refraction were examined as secondary outcomes. STATA 12.0 was used for data extraction and analysis.

**Results:**

A total of 307 eyes were involved in this study to evaluate the effect of atropine on ChT, axial length (AL) and standardized equivalent refraction (SER) in myopic children. Choroidal thickening was significantly higher in the atropine group than in the control group at 1 month (WMD, 6.87 mm, 95% CI, 0.04 to 13.10, *P* = 0.049), whereas it was significantly higher in the atropine group than in the control group at months 6 (WMD, 10.37 mm, 95% CI, −3.21 to 23.95, *P* = 0.135), 12 (WMD, 15.10 mm, 95% CI, −5.08 to 35.27, *P* = 0.143) and at final follow-up (WMD, 11.52 mm, 95% CI, −3.26 to 26.31, *P* = 0.127), the differences were not statistically significant. At months 1 (WMD, −0.03 mm, 95% CI, −0.04 to −0.01, *P* = 0.003), 6 (WMD, −0.07 mm, 95% CI, −0.01 to −0.03, *P* = 0.000), 12 (WMD, −0.13mm, 95% CI, −0.15 to −0.11, *P* = 0.843), and at final follow-up (WMD, −0.08 mm, 95% CI, −0.16 to −0.01, *P* = 0.127), atropine treatment was able to delay the axial elongation. At 1-month follow-up, there was no significant difference in the effect of atropine on SER in myopic children compared with the control group (WMD, 0.01D, 95% CI, −0.07 to 26.31, *P* = 0.127), whereas it was able to control the progression of refractive status at final follow-up (WMD, 11.52 mm, 95% CI, −3.26 to 26.31, *P* = 0.127).

**Conclusion:**

Limited evidence suggests that 0.01% atropine causes choroidal thickening in myopic children at 1 month of treatment. In the short term, choroidal thickness may be a predictor of the effectiveness of atropine in controlling myopia in children. 0.01% atropine is effective in controlling myopic progression in terms of SER and AL.

**Systematic Review Registration:**

http://www.crd.york.ac.uk/prospero, identifier, CRD42022381195.

## Introduction

Myopia is the most common eye disease in the world. In the past few decades, the prevalence of myopia has increased dramatically, becoming a serious worldwide public health problem. It is expected to affect 4.8 billion people worldwide by 2050, meaning that within 30 years, 50% of the world’s population will be myopic. The prevalence rate of myopia in Asia is 80% or higher, which has reached the pandemic level (Dolgin, 2015; Holden et al., 2016; Morgan et al., 2018). At present, low-concentration atropine, orthokeratology, bifocal spectacle lenses, eye exercises, acupuncture and other treatment methods are used to prevent and control myopia. In addition, more outdoor activities and less time spent using electronic screens can also delay the progression of myopia in children (Resnikoff et al., 2008; Lv et al., 2015; Huang et al., 2016; Tay et al., 2017). According to research reports, there is no safe threshold for myopia refractive error, which indicates that there is no “physiological myopia” (Flitcroft, 2012). Once myopia has formed, it can continue to worsen throughout childhood and, in high myopia, even into adulthood (Fricke et al., 2012). Accompanied by high myopia and pathological myopia, changes in the sclera, choroid and retina cannot be prevented by optical correction (Flitcroft, 2012; Morgan et al., 2012; Holden et al., 2016; Morgan et al., 2018). Progressive high myopia has been proven to be a significant risk factor for open-angle glaucoma, cataract, myopic macular degeneration, rhegmatogenous retinal detachment, and myopic choroidal neovascularization, thereby causing irreversible damage to visual acuity (Tideman et al., 2016). Myopia can also affect children’s overall quality of life, especially in terms of academic performance, physical activity, social interaction and future job choices, bringing heavy economic burden (Zhao et al., 2020). The current clinical diagnosis of myopia progression requires prolonged and repeated measurement of refraction and AL, which is not conducive to early assessment of the long-term effectiveness of myopia control programs. Therefore, it is urgent to find efficient measures to control the progression of myopia.

Thinning of the choroid is a significant structural change in myopia. Choroidal thickness (ChT) is considered to be a new and rapid predictor of myopia occurrence and development (Liu et al., 2021; Zhou et al., 2021). Numerous animal studies have shown that the choroid of myopic eyes is thinner than that of normal eyes (Wallman et al., 1995; Troilo et al., 2000; Fitzgerald et al., 2002; Lu et al., 2009). Transient choroidal thickening was present when experimentally induced myopia was successfully inhibited in animals, and was not observed when stimulation was ineffective in preventing myopia (Wallman and Winawer, 2004; Nickla, 2007). Similar changes have also been demonstrated in cross-sectional studies of adults, where the presence of myopia is accompanied by a significantly thinner choroid compared with emmetropia and hyperopia (Howlett and McFadden, 2009; Shin et al., 2012; Wei et al., 2013; Gupta et al., 2015). In children, the choroid contributes more to myopia development than in adults (Smith et al., 2014). It has been reported that the choroid thickness of myopic children and emmetropic children who develop myopia becomes significantly thinner with time, while that of non-myopic children increases significantly with time (Fontaine et al., 2017; Jin et al., 2019). For years, researchers and clinicians have proposed approaches to treat myopia. Atropine, a nonselective muscarinic antagonist, has been widely studied for the prevention and treatment of myopia in children in recent years (Huang et al., 2016). Although little is known about how atropine reduces myopia progression, studies have shown that concentrations ranging from 0.0125% to 1% of atropine can cause choroidal thickening (Ye et al., 2021). The study suggested that 1% atropine increased choroid thickness, while 0.01% atropine decreased choroid thickness after 6 months of treatment (Ye et al., 2020). However, these studies had relatively small sample sizes, and based on their studies, whether and to what extent atropine affects ChT in myopic children is controversial. Given these inconsistent findings, further understanding of the relationship between atropine and ChT is needed, which will allow us to leverage the advantages of atropine to inform future clinical trials. It is generally accepted that meta-analysis is a powerful statistical tool that can overcome the limitations of individual studies with different sample sizes and produce the best estimates. In this study, we aimed to assess whether atropine can thicken the choroid in children with myopia. Therefore, a meta-analysis of all eligible published studies was conducted. We also evaluated the effects of atropine on axial length (AL) and standardized equivalent refraction (SER). To our knowledge, the present study is the first such investigation on this topic.

## Materials and methods

### Search strategy

We performed a comprehensive literature search of articles through the following databases without date limitation: PubMed, EMBASE, and Web of Science databases. The search was updated to 1 June 2023. The following search strategy was performed in PubMed: (((((“Myopia” [Mesh]) OR (Myopias)) OR (Nearsightedness)) OR (Nearsightednesses)) AND (((((((“Choroid” [Mesh]) OR (Sattler Layer)) OR (Choroids)) OR (Sattler’s Layer)) OR (Haller’s Layer)) OR (Haller Layer)) OR (Choriocapillari))) AND ((((((“Atropine” [Mesh]) OR (Atropinol)) OR (Atropine Sulfate)) OR (Atropine Sulfate Anhydrous)) OR (AtroPen)) OR (Atropin Augenol)). The reference list was also checked for relevant articles and the language limit is English.

### Inclusion and exclusion criteria

The primary study outcome was the difference in ChT between the atropine group and the control group at 1 month of treatment, and we examined AL and SER as secondary outcomes. Randomized control trials (RCTs) and cohort studies on the relevant topic were selected according to the following criteria: 1) The participants were school-age children with myopia, aged 6–16 years old were included; 2) Atropine was used in at least one treatment arm; 3) Placebo, single-vision lenses, or other non-atropine treatments were used as controls; 4) The study reported changes in ChT at baseline and 1 month after treatment, with secondary outcomes of mean AL elongation and the change in standardized equivalent refraction (SER); We excluded review articles, case reports, case series, and animal or laboratory studies.

### Data extraction and quality assessment

All candidate articles were evaluated and extracted by two independent authors (Yaqi Yang and Bo Wang). Articles that could not be categorized based on title and abstract alone were retrieved for full-text review. If disagreement occurred, two authors discussed and arrived at consensus with a third author (Lijuan Wei). For each study, the following items were recorded: first author, year of publication, country, total number of cases, baseline ChT, atropine dose, follow-up period, treatment strategy, degree of myopia, and differences in CHT, AL, and SER between the atropine and control groups after 1 month of continuous treatment and at the end of follow-up.

Two authors (Yaqi Yang and Bo Wang) assessed the risk of bias in the included studies using the Cochrane bias risk assessment tool. The rating of bias risk includes six reference factors: random sequence generation, allocation concealment, blinding, incomplete outcome data, selective outcome reporting, and other biases. The results will be graded into three categories: low risk of bias, high risk of bias or unclear risk of bias. If the two authors have any disagreement on the quality of the RCTs, they should discuss and arbitrate with the other author (Wei Zheng).

### Statistical analysis

All extracted data were imported into a database, and all statistical analyses were performed using STATA 12.0 (Stata Corporation, College Station, TX). The weighted mean differences (WMDs) and 95% confidence intervals (CIs) were calculated for each study to assess ChT, AL and SER changes between the two groups (intervention and control). The absolute changes (Means and standard deviations) in ChT, AL and SER were used to calculate the WMDs. Statistical heterogeneity was evaluated using the chi-square-based Q test and the I^2^ statistic. When I^2^ < 50% and *p* ≥ 0.1 indicates no observed heterogeneity, the fixed effects model will be adopted. If there was significant heterogeneity (I^2^ ≥ 50% or *p* < 0.1), a random effects model was used and one-way sensitivity analyses were performed, removing each study in turn to evaluate the influence of each individual trial on the pooled outcomes. Subgroup analysis was performed based on different atropine concentrations, follow-up periods, testing instruments, degree of myopia and treatment. Potential publication bias was evaluated using funnel plots, Begg’s test, and Egger’s test.

## Results

### Search results and study characteristics

**FIGURE 1:**
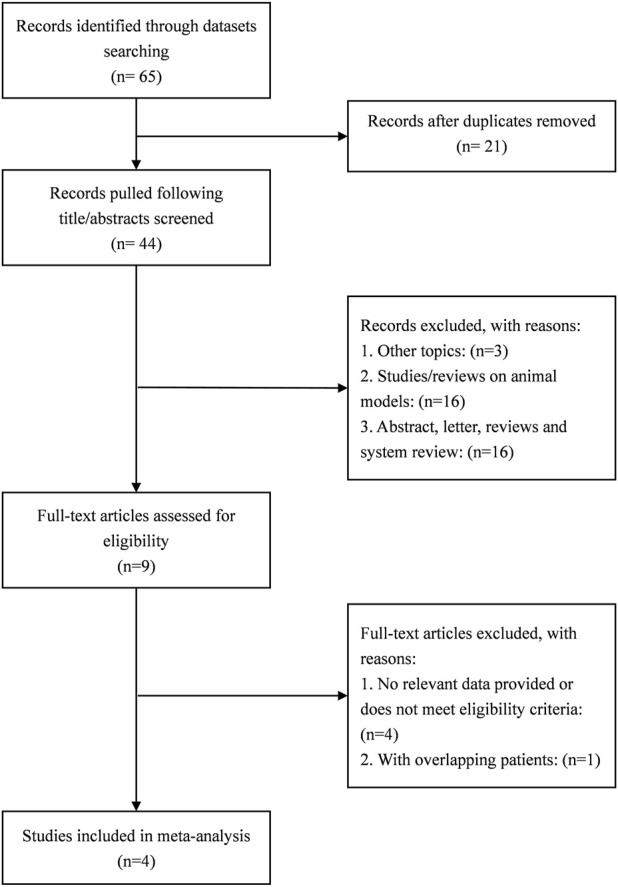
Flow chart of the included studies.

**FIGURE 2:**
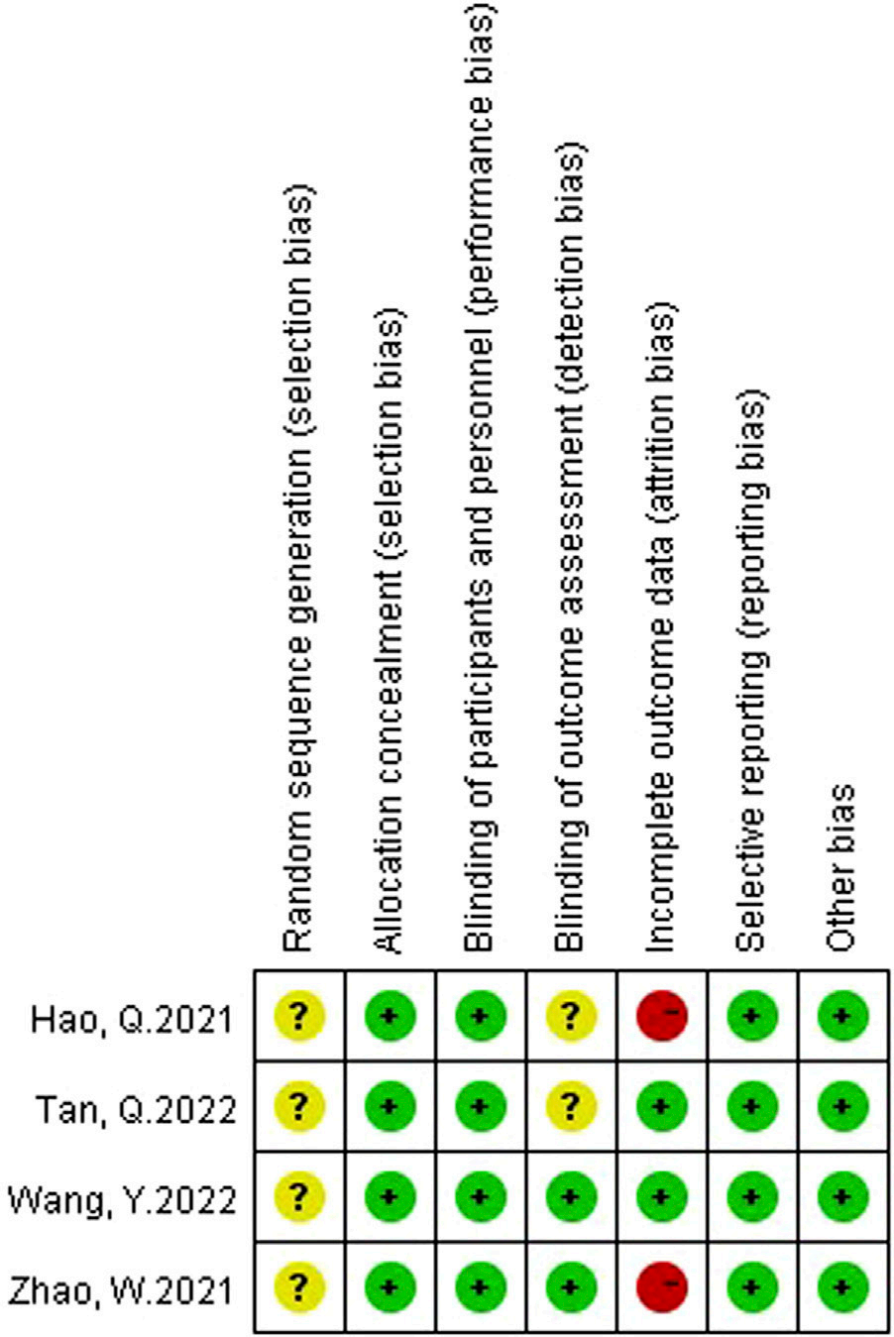
The results of the methodological evaluation.

A total of 65 studies were retrieved. Finally, the data used for the analysis were 4 RCTs, which included a total of five sets of data (Hao and Zhao, 2021; Zhao et al., 2021; Wang et al., 2022; Tan et al., 2023). The literature screening process is shown in Figure 1. The basic characteristics of the 4 studies are shown in the Table 1. There were 157 patients in the atropine group and 150 patients in the control group. 3 studies were carried out on the Chinese mainland and one in Hong Kong. In 3 studies, the atropine group received atropine and orthokeratology, and the control group received orthokeratology. In two other studies, the atropine group used only atropine eye drops, while the control group used placebo or single-vision lenses. All studies included used atropine at a concentration of 0.1%. All studies reported ChT after 1 month of atropine use, and 1 study did not report specific conditions for the AL and SER. Figure 2 reports the quality assessment of included studies according to the Cochrane bias risk assessment tool. There are 2 studies did not report in the form of the causes and management of patients withdrew from the study (Hao and Zhao, 2021; Zhao et al., 2021).

**TABLE 1:** Main characteristics of all the studies included in the meta-analysis.

Study cohort	Year	Country/ Area	No. (T/C)	Experimental baseline ChT Mean(SD), µm	Control baseline ChT Mean(SD), µm	AtropineDose,%	Follow-up, mo	Instrument	Treatment	Control
Hao, Q.	2021	China	21/22	240.64 (19.93)	NA	0.01%	12	NA	atropine+ orthokeratology	orthokeratology
Tan, Q	2022	Hong Kong	34/35	244.20 (46.30)	234.60(47.00)	0.01%	24	Heidelberg Spectralis	atropine+ orthokeratology	orthokeratology
Wang, Y	2022	China	21/18	249.98 (38.26)	230.53(45.53)	0.01%	3	Heidelberg Spectralis	atropine	single-vision lenses
Zhao, W	2021	China	42/37	256.61 (46.55)	258.05(52.34)	0.01%	1	Heidelberg Spectralis	atropine	single-vision lenses
Zhao, W	2021	China	39/36	263.17 (46.55)	266.74(57.50)	0.01%	1	Heidelberg Spectralis	atropine+ orthokeratology	orthokeratology

T, treatment group; C, control group; NA, not available; SD, standard deviation; ChT, choroidal thickness.

### Change in ChT

**FIGURE 3:**
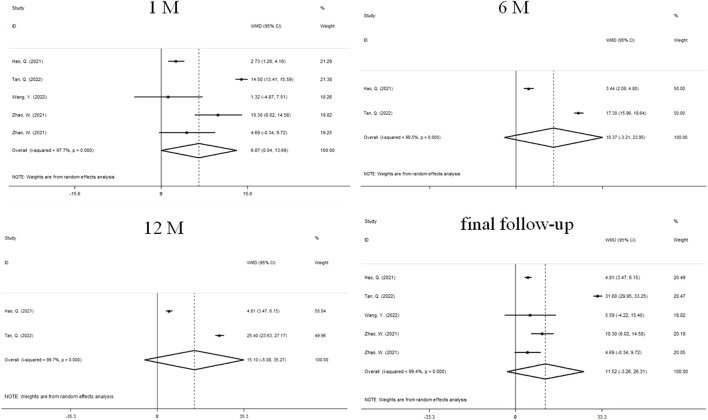
Forest plot of ChT difference between atropine group and control group.

Changes in ChT before and after treatment were reported in 4 studies (Figure 3). There was significant heterogeneity in the study (I^2^ = 97.7%, *P* = 0.000), so we used a random effects model. The combined results showed that after 1 month of treatment, the choroidal thickening degree in atropine group was higher than that in control group, and the combined WMD was 6.87 mm. The difference between the two groups was statistically significant (95%CI: 0.041 to 13.692, *P* = 0.000, Figure 3), while the difference was not statistically significant at 6 months (WMD, 10.37 mm, 95%CI: −3.21 to 23.95, *P* = 0.135), 12 months (WMD, 15.10 mm, 95%CI: −5.08 to 35.27, *P* = 0.143), and final follow-up (WMD, 11.52 mm, 95%CI: −3.26 to 26.31, *P* = 0.127). We performed sensitivity analyses on all included studies to assess whether heterogeneity could be reduced. After the papers were sequentially removed, the results showed a lack of robustness (Supplementary Figures S1, S2). When the treatment period was 1 month, the combined results of the remaining studies were not statistically significant after two sets of data were individually or jointly excluded (Zhao et al., 2021), and no significant heterogeneity was changed under any scenario. The end of follow-up was 1 month for both data sets. At the final follow-up, when the study was removed (Tan et al., 2023), making the results significant and reducing the heterogeneity from substantial to moderate, the study had a follow-up termination time of 24 months. To further explore the sources of heterogeneity, we performed a subgroup analysis (Table 2). We found that at 1 month, differences in treatment and myopia degree and follow-up were not major sources of heterogeneity. At final follow-up, the ChT of atropine-only treatment was higher than that of the control group (WMD, 9.55 mm, 95%CI: 5.63 to 13.47, *P* = 0.029), and the intragroup treatment of atropine-only treatment in addition to Orthokeratology was a source of significant heterogeneity. Additionally, ChT in the atropine group was higher than that in the control group at follow-up ≤ 3 months (WMD, 7.44 mm, 95%CI: 3.42 to 11.48, *P* = 0.029), and intra-group studies at follow-up ≥1 year may be a significant influence on heterogeneity. In contrast, the degree of myopia was not a major source of heterogeneity.

**TABLE 2:** Summary of the meta-analysis results.

Analysis	N	Reference	Random-effects model		Fixed-effects model		Heterogeneity	
			WMD (95%CI)	P	WMD (95%CI)	P	I^2^	Ph
Overall	5	30-33	11.52(-3.26­26.31)	0.127	14.63(13.65­15.62)	0.000	99.40%	0.000
Degree of myopia								
—1.00 to—4.00DS	1	30	4.81(3.47­6.15)	0.000	4.81(3.47­6.15)	0.000	--	--
—1.00 to—6.00DS	3	31,33	15.64(-2.91­34.18)	0.098	26.76(25.29­28.23)	0.000	98.80%	0.000
0.00 to—6.00DS	1	32	5.59(-4.22­15.40)	0.264	5.59(-4.22­15.40)	0.264	--	--
Treatment								
Atropine	2	32,33	9.55 (5.63­13.67)	0.000	9.55(5.63­13.67)	0.000	0.00%	0.389
Atropine+ Orthokeratology	3	30,31,33	13.75(-6.73­34.23)	0.188	14.98(13.96­16.00)	0.000	99.7%	0.000
Follow-up (months)								
≤3	3	32,33	7.44(3.42­11.46)	0.000	7.71(4.62­10.80)	0.000	32.8%	0.000
≥12	2	30,31	18.2(-8.05­44.45)	0.174	15.42(14.38­16.46)	0.000	99.8%	0.000

N, number of studies; WMD, weighted mean differences; 95% CI, 95% confidence interval; Ph, p values of Q test for heterogeneity test. I^2^, I-squared.

### Change in AL

**FIGURE 4:**
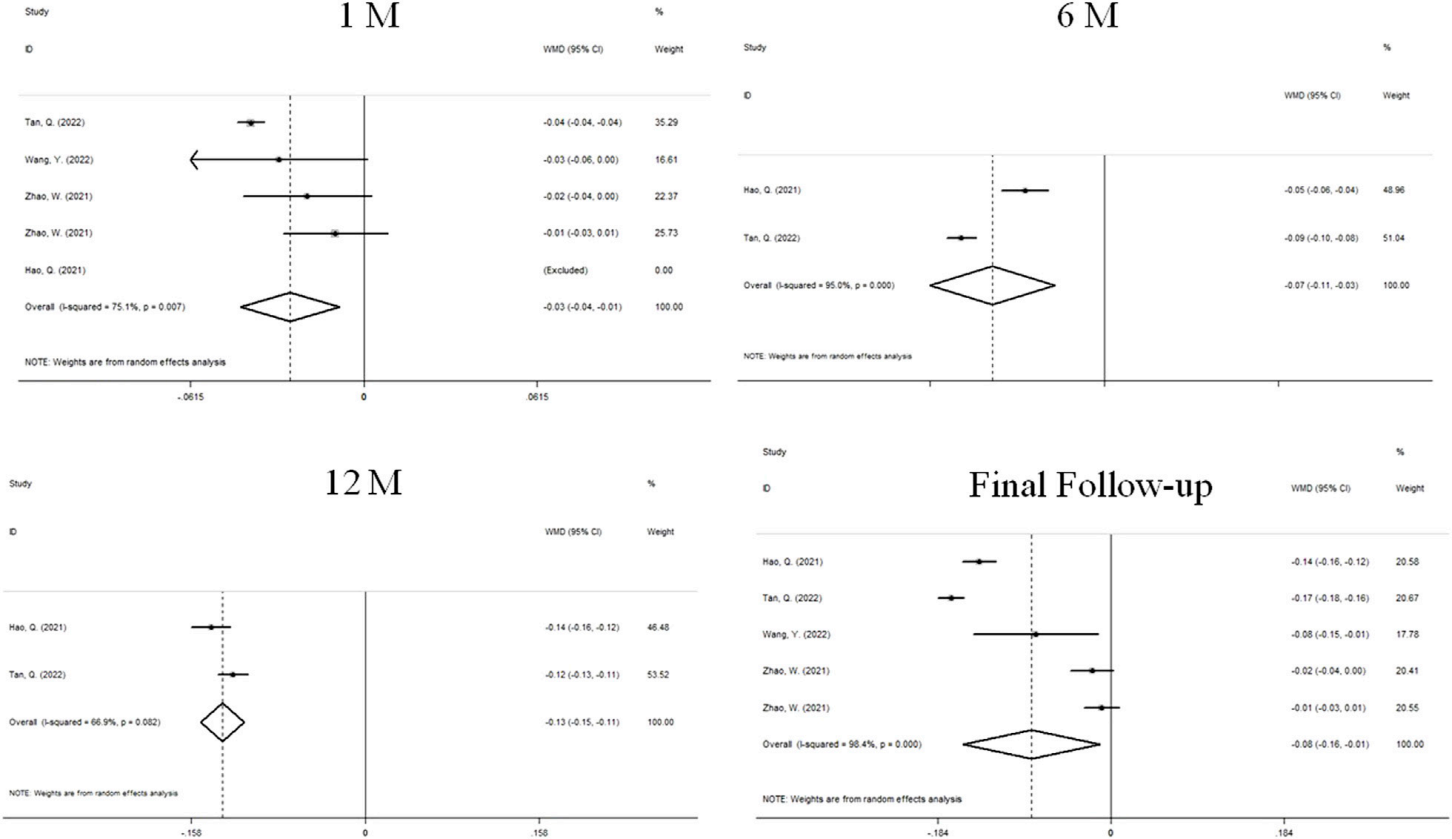
Forest plot of the difference in AL between atropine and control groups.

The changes of AL after treatment were reported in 4 studies (Figure 4). We used a random effects model because the heterogeneity was significant (I^2^ = 75.5%, *P* = 0.007). The combined results showed that AL prolongation in atropine group was significantly lower than that in control group at 1 month of treatment (WMD, −0.03 mm, 95%CI: −0.04 to −0.01, *P* = 0.003), 6 months (WMD, −0.07 mm, 95%CI: −0.11 to −0.03, *P* = 0.000), 12 months (WMD, −0.13 mm, 95%CI: −0.15 to −0.11, *P* = 0.000) and the final follow-up (WMD, −0.08 mm, 95%CI: −0.16 to −0.01, *P* = 0.023), and the difference was statistically significant.

### Change in SER

**FIGURE 5:**
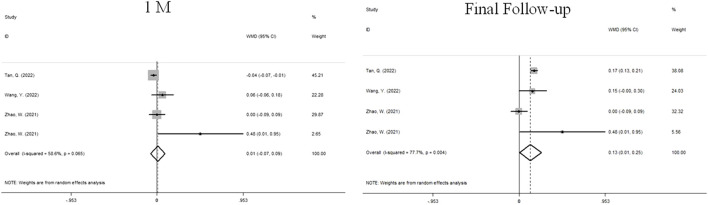
Forest plot of SER difference between atropine group and control group.

Four sets of data from 3 studies analyzed changes in SER (Figure 5). We used a random effects model because the heterogeneity was significant (I^2^ = 58.6%, *P* = 0.065). At 1 month, there was no significant difference between atropine group and control group (WMD, 0.01D, 95%CI: −0.07 to 0.09, *P* = 0.065). At the final follow-up, the children who received atropine showed significantly less progression in refraction than controls (WMD, 0.13D, 95%CI: 0.01 to 0.25, *p* = 0.038).

### Publication bias

Begg’s funnel plot (Supplementary Figure S3) and the Egger’s linear regression test (Supplementary Figure S4) were performed to evaluate publication bias. This study no significant publication bias was detected (*Pr* > |*z*| = 0.540 for Begg’s test and *Pr* > |*t*| = 0.462 for Egger’s test).

## Discussion

In recent years, a large number of studies have examined whether atropine has a choroidal thickening effect and have suggested that ChT is associated with myopia treatment outcomes, but these results are controversial. Therefore, we reviewed the published studies and performed a meta-analysis to more accurately estimate the effect of atropine on ChT in myopia treatment. Based on 4 studies of 307 children, our research led to three main conclusions. Firstly, the results showed that atropine thickened the choroid in myopic children at 1 month of treatment. Atropine continued to have choroidal thickening effects compared with the control group at a follow-up period of ≤3 months, or when excluding a study with a 24-month follow-up period. Secondly atropine was able to significantly slow the growth of the AL at months 1, 6, 12 and at the final follow-up. Thirdly, atropine had no significant effect on SER at the first month, and the progression of refractive status in children treated with atropine was significantly lower than in the control group at the final follow-up.

Although the exact mechanisms of myopia progression are unknown, there is growing evidence pointing to the important role of the choroid in emmetropization (Nickla and Wallman, 2010). Studies have shown that ChT is negatively correlated with AL and that choroidal thinning is higher than that predicted based on passive stretching of the choroid as a result of axial elongation in myopic eyes, and thus changes in ChT may be an active mechanism (Chen et al., 2015). Changes in choroidal thickness may influence scleral remodeling (Nickla and Wallman, 2010). Troilo et al. proposed that the choroid responds to signals from the retina and RPE and may secrete growth factors associated with scleral growth, or that the lateral area of the choroid mechanically influences the area of the sclera and thus the size of the globe (Troilo et al., 2000). A reasonable hypothesis is that a thicker choroid may act as a barrier preventing retinal signals from reaching the sclera, or a thicker choroid may facilitate the diffusion of molecules into the sclera (Nickla and Wallman, 2010). The mechanisms underlying the changes in ChT that accompany myopia have not been elucidated, and the relationship between hypoxia and myopia, as well as the oxygenation function of choroidal blood flow, gives us a basis for hypothesizing that changes in choroidal blood flow may contribute to the changes in ChT (Liu et al., 2021). The effect of atropine on ChT may be related to retinal dopamine release. Intravitreal injection of 250 µg of atropine was able to increase dopamine release from the retina of chickens (Mathis et al., 2020), in addition the same dose of atropine prevented the development of form deprivation myopia (Diether et al., 2007). Mathis et al. found that dopamine antagonism resulted in a reduction of atropine-induced choroidal thickening, and that the higher the level of retinal dopamine, or release, the thicker the choroid was Mathis et al. (2023). Previous experimental studies have shown that atropine may induce nitric oxide (NO) synthesis and release, which in turn affects changes in ChT (Carr and Stell, 2016; Mathis et al., 2020). The mechanism of ChT changes may involve signals such as dopamine, acetylcholine and NO. In addition, glucagon, insulin and the VEGF family can also modulate choroidal thickness (Nickla and Wallman, 2010; Zhang and Wildsoet, 2015).

Currently, atropine is the most effective and widely used drug used to treat myopia in humans (Pineles et al., 2017; Wildsoet et al., 2019). Previous studies have clearly demonstrated the presence of myopic rebound with medium to high doses of atropine. Low doses of atropine are effective in slowing myopia progression, have fewer side effects with less rebound effect, and are sufficiently safe (Chia et al., 2012; Chia et al., 2014; Chia et al., 2016; Pineles et al., 2017). Some scientists hypothesize that the effective site of atropine may be in the choroid (Kolb et al., 1995; Zhang et al., 2016; Chiang and Phillips, 2018). Atropine treatment has been shown to increase the resting thickness of the choroid in animals and humans (Nickla et al., 2013; Zhang et al., 2016), and evidence from chick model suggests that atropine may act directly on the choroid (Nickla et al., 2013). Zhang et al. showed that the use of 1% atropine gel twice daily for 1 week resulted in an increase in resting ChT in healthy children (Zhang et al., 2016). After a single topical application of 0.01% atropine, the intraocular concentration of atropine may reach the choroidal extent within 60 min, which in the short term may not only inhibit the reduction of CHT in humans caused by hyperopic defocus, but also directly thicken the choroid in myopic adults (Sander et al., 2019). In a randomized clinical study of myopia in children conducted by Tan et al. (2023) 0.01% atropine in combination with orthokeratology was found to have better choroidal thickening compared to orthokeratology alone. In addition, based on a 3-year study of low concentrations of atropine to control myopic progression, Yam et al. suggested that low concentrations of atropine were able to thicken the choroid in myopic children, albeit in a concentration-dependent relationship. Changes in ChT can be used to assess the outcome of long-term treatment of myopia (Yam et al., 2022). There are also a series of studies that contrast with the conclusions of the above studies. A randomized controlled trial by Hannan et al. showed that 1% atropine caused choroidal thickening while 0.01% atropine had little to no choroidal thickening. Myopic rebound and reduced choroidal changes occurred after 1% atropine was changed to 0.01% atropine (Xu et al., 2023). Yuliang et al. concluded in a RCT with a 3-month follow-up period that the use of 0.01% atropine resulted in an increase in ChT, but the difference was not statistically significant when compared to the regular single-vision lenses group (Wang et al., 2022). Wu et al. (2023) demonstrated that the administration of a 0.01% atropine dose for 3 months can lead to an increase in ChT in myopic children, with continued thickening observed up to 12 months .In addition to this, a recent meta-analysis showed that 0.01% atropine, did not show an increase in the choroid (Meng et al., 2023).

These different findings may be attributed to inconsistencies in the time points of follow-up and differences in atropine concentrations. Unfortunately, there are no studies of changes in CHT after the use of different doses of atropine with continuous follow-up at multiple time points.

Presently, assessing the effectiveness of interventions for myopia requires repeated measurements of changes in AL and refraction, which is not conducive to early intervention and management of myopia, and thus we expect ChT to be able to predict the effectiveness of these interventions. In this study, 0.01% atropine was shown to have a choroidal thickening effect in the early stages of use (at 1 month of treatment), and continued to cause choroidal thickening at a follow-up period of ≤3 months. In summary, at 1 month, 0.01% atropine caused choroidal thickening in myopic children. The short-term response in ChT can be used to assess long-term outcomes in myopia prevention and control. More plausible and relevant future studies are needed to support this conclusion.

This meta-analysis has several limitations. First of all, there was heterogeneity in the difference in ChT, and we performed relevant sensitivity analyses and subgroup analyses but still could not fully derive the source of the heterogeneity. We should therefore be cautious about the interpretation of the results. Second, there are few studies that fulfill the screening conditions, which may lead to publication bias that could not be detected by Begg’s and Egger’s tests and limit the generalizability of the findings. Third, the inclusion and exclusion criteria of this meta-analysis provided limited information on study characteristics, so the quality and applicability of included studies need to be further improved. Fourthly, there was no statistical difference in the combined results of this study on the long-term effects of atropine on ChT in myopic children. It is expected that more comprehensive data can be collected in the future to support our conclusions and enrich the clinical significance of the results of this study. Finally, the language of this study was limited to English, all studies focused on Asian populations, and only studies using atropine at a concentration of 0.01% met the inclusion criteria, so the possibility of publication bias cannot be completely ruled out.

## Data Availability

The original contributions presented in the study are included in the article/Supplementary Material, further inquiries can be directed to the corresponding authors.

## Supplementary material

SUPPLEMENTARY FIGURE S1 Sensitivity analysis of papers documenting ChT changes (1 month).

SUPPLEMENTARY FIGURE S2 Sensitivity analysis of papers documenting ChT changes (final follow-up).

SUPPLEMENTARY FIGURE S3 Begg’s funnel plot.

SUPPLEMENTARY FIGURE S4 Egger’s linear regression test.
