# Supplementary figures and images for: Effects of atropine on choroidal thickness in myopic children: a meta-analysis
Source: Front Pharmacol. 2024 Oct 21;15:1440180. doi: 10.3389/fphar.2024.1440180 (PMC11533146; doi:10.3389/fphar.2024.1440180)

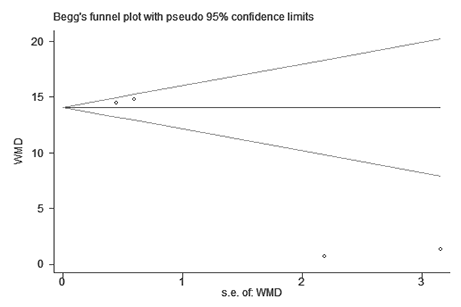

Supplement: Supplementary file 1 [file Image3.tif]

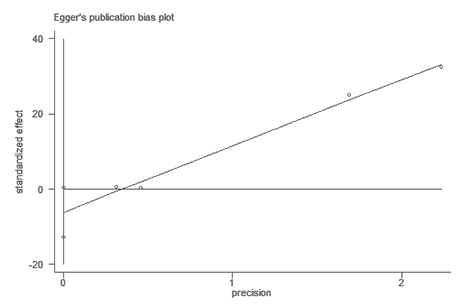

Supplement: Supplementary file 2 [file Image4.tif]

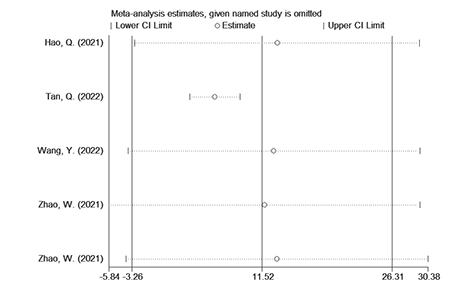

Supplement: Supplementary file 3 [file Image2.tif]

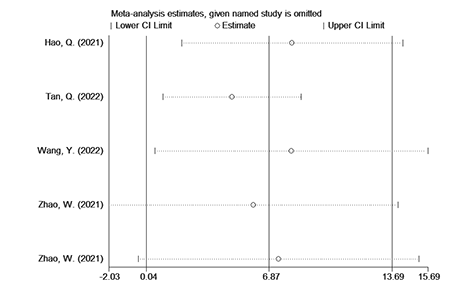

Supplement: Supplementary file 4 [file Image1.tif]
